# Exploring Drugs and Vaccines Associated with Altered Risks and Severity of COVID-19: A UK Biobank Cohort Study of All ATC Level-4 Drug Categories Reveals Repositioning Opportunities

**DOI:** 10.3390/pharmaceutics13091514

**Published:** 2021-09-18

**Authors:** Yong Xiang, Kenneth Chi-Yin Wong, Hon-Cheong So

**Affiliations:** 1 Lo Kwee-Seong Integrated Biomedical Sciences Building, School of Biomedical Sciences, Faculty of Medicine, The Chinese University of Hong Kong, Shatin, Hong Kong, China; xyong11@link.cuhk.edu.hk (Y.X.); mail@cywong.hk (K.C.-Y.W.); 2 KIZ-CUHK Joint Laboratory of Bioresources and Molecular Research of Common Diseases, Kunming Institute of Zoology, Kunming 650223, China; 3 CUHK Shenzhen Research Institute, Shenzhen 518172, China; 4 Department of Psychiatry, Faculty of Medicine, The Chinese University of Hong Kong, Shatin, Hong Kong, China; 5 Margaret K.L. Cheung Research Centre for Management of Parkinsonism, The Chinese University of Hong Kong, Shatin, Hong Kong, China; 6 Brain and Mind Institute, The Chinese University of Hong Kong, Shatin, Hong Kong, China; 7 Hong Kong Branch of the Chinese Academy of Sciences Center for Excellence in Animal Evolution and Genetics, The Chinese University of Hong Kong, Shatin, Hong Kong, China

**Keywords:** COVID-19, drug repositioning, UK Biobank, vaccine

## Abstract

Effective therapies for COVID-19 are still lacking, and drug repositioning is a promising approach to address this problem. Here, we adopted a medical informatics approach to repositioning. We leveraged a large prospective cohort, the UK-Biobank (UKBB, *N* ~ 397,000), and studied associations of prior use of all level-4 ATC drug categories (*N* = 819, including vaccines) with COVID-19 diagnosis and severity. Effects of drugs on the risk of infection, disease severity, and mortality were investigated separately. Logistic regression was conducted, controlling for main confounders. We observed strong and highly consistent protective associations with statins. Many top-listed protective drugs were also cardiovascular medications, such as angiotensin-converting enzyme inhibitors (ACEI), angiotensin receptor blockers (ARB), calcium channel blocker (CCB), and beta-blockers. Some other drugs showing protective associations included biguanides (metformin), estrogens, thyroid hormones, proton pump inhibitors, and testosterone-5-alpha reductase inhibitors, among others. We also observed protective associations by influenza, pneumococcal, and several other vaccines. Subgroup and interaction analyses were also conducted, which revealed differences in protective effects in various subgroups. For example, protective effects of flu/pneumococcal vaccines were weaker in obese individuals, while protection by statins was stronger in cardiovascular patients. To conclude, our analysis revealed many drug repositioning candidates, for example several cardiovascular medications. Further studies are required for validation.

## 1. Introduction

Coronavirus disease 2019 (COVID-19) has resulted in a pandemic affecting more than a hundred countries worldwide [1,2,3]. More than 220 million confirmed infections and 4.56 million fatalities have been reported worldwide as of 6 September 2021 (https://coronavirus.jhu.edu/map.html, accessed on 6 September 2021). Besides the burden due to the disease itself, COVID-19 has created heavy burdens on the medical systems in many countries and has led to delays in the diagnosis and treatment of other types of diseases [4,5]. Therefore, it is of urgent public interest to gain deeper understanding into the disease, including identifying risk factors (RFs) for infection and severe disease, and uncovering new treatment strategies.

Although vaccines have been developed for COVID-19, its distribution is highly uneven and only a small proportion of the world’s population has been fully vaccinated so far. In addition, vaccine hesitancy remains a major issue that has led to suboptimal vaccination coverage [6,7]. Inadequate knowledge and awareness of COVID-19, especially among the younger population, may also contribute to the continuous rise in the number of cases [8]. Coupled with viral variants that may be associated with increased transmission and reduced vaccine effectiveness [9], the search for drugs that may reduce susceptibility to disease and/or disease severity remains highly important.

A number of clinical risk factors (e.g., age, obesity, cardiometabolic disorders, renal diseases, presence of multiple comorbidities) [10,11,12,13,14,15] have been found to increase the risk of infection or complications. However, it is less well-known how different drugs may affect the risks of COVID-19 or its severity. Importantly, drugs with protective effects may be potentially repurposed for the prevention or treatment of the disease, as development of a new drug is often extremely lengthy and costly.

Drug repositioning by computational or statistical approaches for COVID-19 is an area of intense interest. Please refer to other reviews (e.g., [16,17,18]) for an overview of recent studies. For instance, one widely used methodology is the network-based approach, which can integrate different data sources, including omics data and drug–protein–disease interaction networks [16,19,20,21]. Another methodology is the structure-based approach, which enables a large number of compounds to be screened for their ability to bind to known or predicted molecular targets for COVID-19 treatment [16,22,23,24,25]. These methodologies are promising but may have their limitations. For example, they generally do not provide direct evidence for the candidates’ effectiveness in real-world or clinical settings. In addition, these approaches may be limited by inadequate knowledge of the pathophysiology and molecular basis of COVID-19. Another limitation is that most drug repositioning studies did not consider patient characteristics; for example, a drug may be more effective within a certain age group or in those with a certain comorbidity. In addition, the effect size (e.g., relative risk reduction) of individual drugs and the level of statistical significance usually cannot be easily estimated by network/structure-based approaches.

Here, we employed a different methodology *not* previously applied to drug repositioning studies for COVID-19. We adopted a medical informatics approach which involves screening a large number of drugs for their associations with the disease, leveraging a large-scale population cohort. In brief, we performed a comprehensive study on all Anatomical Therapeutic Chemical Classification System (ATC) level-4 drug categories (*N* = 819) and assessed their associations with susceptibility to, and severity of, COVID-19 in the UK Biobank (UKBB), controlling for possible confounders. Vaccines were also included for analysis. To our knowledge, this is the most comprehensive analysis to date to screen for drug associations and repositioning candidates for COVID-19, leveraging real-world population data.

While pharmacoepidemiology studies are typically focused on one or a few drugs, COVID-19 is a new disease, and we still have limited understanding of its pathophysiology and treatment. As a result, a hypothesis-driven approach may have important limitations of missing potential drug associations and new repositioning candidates. In the field of genetic epidemiology, it has been observed that hypothesis-driven candidate gene studies are not as reliable as genome-wide association studies (GWAS) [26] which are relatively unbiased, indicating merits of the latter approach. In the same vein, here we adopted a “drug-wide” association study approach, which provides a systematic and unbiased assessment of drug associations and repositioning candidates. This approach has also been advocated before [27].

In the present study, we performed rigorous analyses on the impact of medications/vaccinations on the risk of infection, disease severity, and mortality. Analyses were also conducted within infected patients, tested subjects, and the whole population respectively, and for five different time windows of prescriptions. We also performed further subgroup and interaction analyses to reveal differential effects of the drugs in people with different clinical background. This may enable more “personalized” drug repositioning, i.e., prioritizing drug candidates for specific patient subgroups.

## 2. Methods

### 2.1. UK Biobank Data

The UK Biobank is a large-scale prospective cohort comprising over 500,000 subjects aged 40–69 years who were recruited in 2006–2010 [28]. In this study, subjects with recorded mortality before 31 January 2020 (*N* = 28,930) were excluded, as it was the date for the first recorded case in UK. This study was conducted under project 28732.

### 2.2. COVID-19 Phenotypes

COVID-19 outcome data were downloaded from UKBB data portal. Information regarding COVID-19 data in the UKBB can be viewed at http://biobank.ndph.ox.ac.uk/showcase/exinfo.cgi?src=COVID19 (accessed on 3 November 2020). Briefly, the latest COVID test results were downloaded on 6 November 2020 (last update 3 November 2020). We consider inpatient (hospitalization) status at testing as a proxy for severity. Data on date and cause of mortality were also extracted (latest update on 21 October 2020). Cases indicated by U07.1 were considered to be (laboratory-confirmed) COVID-19-related fatalities.

A case was considered as having “severe COVID-19” if the subject was hospitalized and/or if the cause of mortality was U07.1. We required both test result and origin to be 1 (positive test and inpatient origin) to be considered as a hospitalized case. For a small number of subjects with initial outpatient origin and positive test result, but changed to inpatient origin and negative result within 2 weeks, we still considered these subjects inpatient cases (i.e., assume the hospitalization was related to the infection).

For a minority of subjects (*N* = 19) whose mortality cause was U07.1 but test results were negative within one week, to be conservative, they were excluded from subsequent analyses.

### 2.3. Medication Data

**Figure 1:**
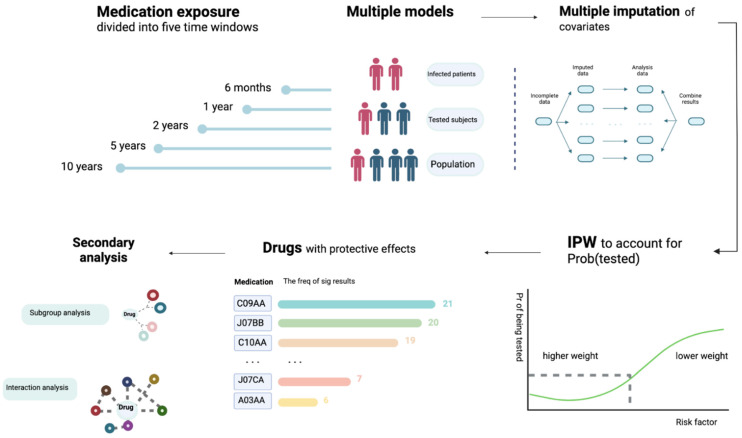
An overview of the analytic workflow. We considered five exposure time windows and multiple statistical models. We conducted analyses within infected patients, tested subjects, and the whole population, respectively. Effects of prescribed medications/vaccinations on the risk of infection, severity of disease (hospitalization as proxy) and mortality were investigated separately. Missing data were accounted for by multiple imputation. Inverse probability weighting (IPW) of the probability of being tested (Prob(tested)) was employed to reduce testing bias. Multivariable logistic regression was conducted, controlling for main confounders. We primarily focused on drugs with protective effects, as residual confounding tends to bias towards harmful effects. In addition, we performed further subgroup and interaction analysis to identify factors that may modify the drug effects.

Medication data was obtained from the primary care data for COVID-19 research in UKBB (details available at https://biobank.ndph.ox.ac.uk/showcase/showcase/docs/gp4covid19.pdf, accessed on 9 November 2020). We made use of the latest release of General Practice (GP) records released by UKBB, which contains prescription data from two electronic health record (EHR) systems (TPP or EMIS) for ~397,000 UKBB participants. The drug code and issue date of each drug are available. Please also refer to Figure 1 for an overview of our analysis workflow.

#### 2.3.1. Time Window of Prescriptions

Since the GP records cover many years of prescriptions, we set time windows to restrict prescriptions with a certain time period as the “exposure”. The “index date” was defined as (1) the date of the first positive COVID-19 test for infected subjects (for U07.1 cases, the mortality date was regarded as the index date if no test record was found); or (2) the date of last test for those who were tested negative; or (3) 3 November 2020 (the date of the latest update of COVID-19 test results) for those who were untested.

The issue date of each prescription was available, but the duration was not. Time windows were determined by whether the drug was issued within a specified period before the index date. The following windows were considered for medications: 6 months, 1 year, 2 years, and 5 years. Narrower time windows (<6 months) may not be desirable and may lead to many prescriptions being missed, as the latest issue date was 25 July 2020, but the latest index date was 3 November 2020.

As for vaccines, unlike many medications, vaccines are not prescribed regularly, and most vaccines only need to be given once or less than a few times; hence, a narrow time window is not optimal due to sparsity of data. For seasonal vaccines, namely flu vaccines, they are usually given in autumn (September to November) or early winter in the UK. A time window of 6 months will lead to missing most of the flu vaccines given. On the other hand, it is also reasonable to consider a longer time window (e.g., 10 years) as vaccine effects can be more long-lasting [29]. In view of the above, we considered time windows of 1, 2, 5, and 10 years for vaccinations. For flu vaccines, we defined “past 1 year” as prescriptions from 1 September 2019 onwards (and similarly for past *k* years) to account for the seasonal nature of vaccination.

#### 2.3.2. Mapping to ATC

All the medications were mapped to the ATC Classification (https://www.genome.jp/kegg-bin/get_htext?br08303, accessed on 9 November 2020). Drug categories were defined by the fourth level of ATC classification.

### 2.4. Covariate Data

We performed multivariable regression analysis with adjustment for potential confounders including basic demographic variables (age, sex, ethnic group), comorbidities (coronary artery disease (CAD), diabetes (DM), hypertension, asthma, chronic obstructive pulmonary disease (COPD), depression, dementia, history of cancer, blood urea and creatinine reflecting renal function), indicators of general health (number of medications taken, number of non-cancer illnesses), anthropometric measures (body mass index (BMI)), socioeconomic status (Townsend deprivation index) and lifestyle risk factor (smoking status). For disease traits, we included information from ICD-10 diagnoses (code 41270) and self-reported illnesses (code 20002), and incorporated data from all waves of follow-ups. Subjects with no records of the relevant disease from either self-report or ICD-10 were regarded as having no history of the disease.

### 2.5. Sets of Analysis

We performed a total of eight sets of analysis (Table 1). The impact of prescribed medication/vaccination on the risk of infection (Models E and F), severity of infection (Models A, C, and G) and risk of mortality (Models B, D, and H) from COVID-19 were investigated separately. Both hospitalized and fatal cases were grouped under the “severe” category.

We also considered different study designs and conducted our analyses with different comparison samples. Models A and B are restricted to the infected subjects, while models C, D, and E involve comparison of severe, fatal and general infected cases to the general population (with no known diagnosis of COVID-19). On the other hand, models F, G, and H compared infected, severe, and fatal cases, respectively, against subjects who were tested negative for SARS-CoV-2.

There were 397,000 subjects in the UKBB with available GP prescription records. Among them, 30,835 subjects have received at least one COVID-19 test, and 3858 had been tested positive. There were 1318 cases classified as “severe” (hospitalized or mortality from COVID-19) and 170 fatal cases. In total 393,142 UKBB participants did not have a known diagnosis of COVID-19. The detailed count of participants for each model is listed in Table 2.

### 2.6. Statistical Analysis Methods

Logistic regression (using the R package speedglm) was used to examine the impact of medication on different outcomes in the eight sets of analysis. For more stable estimates, analysis was not performed if the number of subjects taking the drug in the affected or unaffected group was less than five. All statistical analyses were conducted using R. The false discovery rate (FDR) approach by Benjamini and Hochberg [30] was performed to control for multiple testing. This approach controls the expected proportion of false positives among the rejected null hypotheses.

### 2.7. Imputation of Missing Data

Missing values of remaining features were imputed with the R package “missRanger”. The program is based on missForest, which is an iterative imputation approach based on random forest (RF). It has been widely used and shown to produce low imputation errors and good performance in predictive models [31]. The program missRanger is largely based on the algorithm of missForest, but uses the R package “ranger” [32] to build RF for improvement in speed (we found that other packages, such as MICE and missForest, are computationally too slow to produce results for the large-scale analyses here). Predictive mean matching (pmm) was employed to avoid imputation of values not present in the original data, and to increase variance to more realistic levels for multiple imputation (MI). We followed the default settings with pmm.k = 5 and num.trees = 100. We performed the analyses on multiply imputed datasets (imputed for 10 times) and combined the results by Rubin’s rules [33] using the function “mi.meld” under the R package “amelia”. Another advantage of missRanger is that out-of-bag errors (in terms of classification errors or normalized root-mean-squared error) could be computed, which provides an estimate of imputation accuracy.

### 2.8. Inverse Probability Weighting of the Probability of Being Tested

Bias due to non-random testing has been discussed previously in other works [34,35]. As a person has to be tested to be diagnosed with COVID-19, factors leading to increased probability of being tested will also lead to an apparent increase in the risk of infection [35]. In addition, it has been raised that collider bias can occur when conditioned on the tested group. This could result in spurious associations, for example, between a risk factor and COVID-19 severity if both increases the probability of being tested (Pr(tested)). One way to reduce this kind of bias is to employ inverse probability weighting (IPW) of Pr(tested). Essentially, we wish to create a pseudo-population, or mimic a scenario under which testing is random instead of selected for certain subgroups. The IPW approach up-weighs those who are less likely to be tested and down-weighs those who have a high chance of being tested. This may create more unbiased estimates of the effects of drugs.

We took reference to the approach described in [34] to analyze the data with IPW. Following our recent work [36] which aims to predict COVID-19 severity with machine learning (ML), here we also employed an ML model (XGboost) to predict Pr(tested) based on a range of factors. An advantage of using ML models is that nonlinear and complex interactions can be considered, which may improve predictive performance over logistic models. We employed the same set of predictors as in our previous work [36], and followed the same analysis strategy of hyper-parameter tuning and cross-validation to obtain predicted probabilities (please refer to [36] for details). Beta-calibration [37] was performed, and the resulting average AUC was 0.622. The predicted probabilities (i.e., Pr(tested)) were used to construct weights for IPW. Stabilized weights [38] were used.

### 2.9. Subgroup Analysis

For selected drugs showing tentative protective effects, we also performed further subgroup and interaction analyses. These drugs included cardiovascular medications listed in Table 3, four vaccines with protective associations (influenza, pneumococcal, typhoid, and combined bacterial/viral vaccines), and other top drugs with consistent protective associations across multiple models/time windows as listed in Table 4.

Subgroup analysis was performed with respect to main demographic features (age, sex, and ethnicity) and main comorbidities (same as the diseases listed under “covariate data”). We also compared log(OR) estimates across the subgroups with or without the risk factor of interest. The test statistic was obtained by $z=\left(\beta_{1}-\beta_{2}\right)/\sqrt{var\left(\beta_{1}\right)+var\left(\beta_{2}\right)}$, where $\beta_{1}$ and $\beta_{2}$ refer to the coefficients under the two independent subgroups.

### 2.10. Interaction Analysis

As a complementary approach, we also performed analysis with a logistic model including an interaction term (drug*risk_factor). The same set of drugs and risk factors were studied. The two approaches are similar in principle; however, stratified analysis yields more unbiased estimates if confounders have subgroup-dependent associations, while the interaction term approach produces more precise (lower-SE) estimates (hence higher power to detect interactions) [39].

### 2.11. Controlling for Other Drugs

We also performed additional regression analyses controlling for other top-ranked drugs. Two sets of analyses were conducted. In the first set of analysis, we controlled for the top 10 or 20 protective and harmful drugs in each time window and model. As for the second analysis, for drugs with protective associations, we controlled all other protective drugs with FDR < 0.05 or 0.1 (this analysis was performed for protective drugs only, as there were too many drugs associated with harmful effects to be included as covariates).

## 3. Results

Due to the large number of models and drugs being studied, we highlight the main results and findings from different sensitivity analysis.

Confounding by indication and other comorbidities is unavoidable, and, in particular, drugs showing harmful effects may possibly be explained by such confounding. On the other hand, as it is expected that most diseases tend to *increase* the risk/severity of infection, drugs showing *protective* effects are much less likely to be affected by confounding, and such associations may be relatively more reliable. We therefore place a greater emphasis on protective drugs in the sections below; this is also in line with our primary objective to prioritize repositioning candidates. Drugs with harmful effects are briefly discussed for comprehensiveness.

A summary of the demographic and covariate data of the original UKBB dataset is shown in Table S1. The missing rates and out-of-bag (OOB) errors for different variables from multiple imputations are shown in Table S2.

### 3.1. Primary Analysis with Multiple Imputation of Covariates

Full results of all drug categories across all time windows (including 6, 12, 24, 60, and 120 months; the last time window only for vaccines) are shown in Tables S6–S10. All protective associations (with at least nominal significance, i.e., *p* < 0.05) are shown in Table S3, while all association results with vaccines are presented in Table S4. For drugs associated with increased odds of infection/severity, we also summarize the top 10 drugs (ranked by *p*-value) from each model and time window, and organize them together in Table S5.

#### 3.1.1. Overview

Across all categories, statins showed the strongest and most consistent protective associations. Highly significant protective effects were seen across infected subjects, tested subjects, or the whole population, especially in reducing the severity or mortality of infection. Albeit with smaller effect sizes, we also observed that statins might be linked to lower susceptibility to infection (model E). Interestingly, a number of top-listed drugs are also cardiovascular medications, such as angiotensin-converting enzyme inhibitors (ACEI), angiotensin receptor blockers (ARB), calcium channel blocker (CCB), and beta-blockers.

For simplicity, odds ratios (OR) are presented for a time horizon of 1 year if not further specified.

#### 3.1.2. Drugs for Cardiometabolic Disorders

Significant protective associations with FDR < 0.05 are shown in Table 3. Statins showed protective effects across models A, C, D, E, and G. Significant protective effects against severe infection were seen among infected subjects (OR for prescriptions within a 12-month window, same below: 0.50, 95% CI: 0.42–0.60), tested subjects (OR = 0.63, 0.54–0.73), or when comparing severe cases to the general population (OR = 0.49, 0.42–0.57). In addition, protective association against fatal infection was observed (OR = 0.51, CI 0.34–0.74). Statins was also associated with lower susceptibility to infection, with ORs of 0.83 (CI: 0.77–0.91) and 0.86 (CI: 0.79–0.93) for prescriptions within 1 year and 2 years, respectively.

Another group of drugs with highly consistent protective associations were *ACEI and ARB*. ACEI showed protective associations against severe disease among infected subjects (model A: OR for 1-year time window, same below: 0.68, CI: 0.54–0.86), and when compared to the general population (model C: OR 1 year = 0.61, CI: 0.51–0.74) or test-negative subjects (model G: OR 1 year = 0.71, CI: 0.59–0.85). We also observed association with lower odds of infection at a population level (model E: OR 1 year = 0.81, CI: 0.73–0.90); the effect size seemed to decrease over longer time windows. ARBs also showed protective associations against severe disease in the population (model C: OR 1 year = 0.68, CI: 0.54–0.85) or among tested individuals (model G: OR 1 year = 0.68, CI: 0.55–0.87).

Biguanides (mainly metformin) were associated with lower odds of severe illness among the infected (model A: OR for 2-year time window = 0.60, CI: 0.42–0.86) and in the population (model C; OR 1 year = 0.67, CI: 0.51–0.88). Other drugs of interest include beta-blockers, which were associated with lower risk of infection among tested subjects (model F, OR 1 year = 0.80, CI: 0.70–0.91), and CCBs (C08CA) which were associated with lower odds of severe disease in the population (model C, OR 1 year: 0.76, CI: 0.64–0.90).

#### 3.1.3. Vaccines

Significant associations for vaccines with FDR < 0.05 are shown in Table 5. One of the most consistent associations was observed for influenza vaccines. Protective associations were observed across almost all models (B to H), and across all time windows. Flu vaccination was associated with lower odds of infection when compared to population controls (model E; OR 1 year = 0.73, CI: 0.65–0.83) or compared to test-negative individuals (model F; OR 1 year = 0.60, CI: 0.53–0.68). Similar protective effects were also observed when restricting the cases to severe cases (model C: OR 1 year = 0.74; CI: 0.60–0.91; model G: OR 1 year = 0.61, CI: 0.50–0.76). Association with lower odds of mortality was also observed, although the confidence interval is wide as the number of fatal cases was small (model D: OR 1 year = 0.28, CI: 0.13–0.63; model H: OR 1 year = 0.23, CI: 0.11–0.52). The effect sizes in general became weaker with longer time windows.

In view of the significant findings, we repeated the analyses on flu vaccines with other ways to define the exposure (Table S14). First, we defined the exposure based on the actual season of vaccination instead of any vaccines received in the past *k* years. For people who had received flu vaccination in 2019–2020 (regardless of vaccination in other years), the OR for infection was 0.60 (CI: 0.53–0.68), compared to those who had not (test-negative subjects as controls, model F; same below). The OR was attenuated to 0.76 (CI: 0.67–0.87) if the exposure was defined as flu vaccination in 2015–2016 (regardless of vaccination in other years). We then narrowed down the exposure as receiving flu vaccine in the last season (2019–2020) but *not* in 2018–2019; the resulting OR was 0.67 (CI: 0.53–0.83). On the other hand, if we considered exposure as vaccination in 2018–19 but not 2019–20, the OR became weaker and nonsignificant (OR = 0.80, CI: 0.63–1.01). Those who received the vaccine consecutively for the last two seasons had similar but slightly stronger protection from infection (OR = 0.59, CI: 0.51–0.69); however, the CI overlaps with other estimates. A similar pattern of association was observed for model E (population controls). In general, more recent vaccination was associated with stronger protective effects.

Pneumococcal vaccines were also associated with protection against infection, especially within tested subjects (model F: OR 1 year = 0.50, CI: 0.31–0.82), which shows a trend of attenuation with longer time windows (OR for 10-year window = 0.67, CI: 0.51–0.87). Another group of vaccines showing protective effects is J07CA (bacterial and viral vaccines), which was significant under model F (OR for 1-year window: 0.56, CI: 0.38–0.84); it also showed weakening of effect over time. Other significant associations included tetanus and typhoid vaccines, which were observed to be protective against infections.

#### 3.1.4. Other Drugs Showing Protective Associations

Significant results for other drugs having protective effects and FDR < 0.05 are shown in Table 6. As for other drugs, proton pump inhibitors (PPI) were associated with lower odds of infection when we compared test-positive against test-negative patients (model F: OR 1 year = 0.77, CI: 0.71–0.83); the ORs showed a gradient with largest effect within 6 month of use (OR = 0.72) and became weaker at the 5-year time window (OR = 0.87). PPI was also significantly associated with lower severity of disease.

Natural and semisynthetic estrogens (ATC G03CA) were linked to lower risk of infection and severity in the tested population (model F: OR 1 year = 0.67, CI: 0.58–0.78), which showed attenuation of effect over time. The largest effect size was noted within 6 months of use (OR = 0.63), which was attenuated for a 5-year time window (OR = 0.73). Similar protective associations were observed under model G, with severity as the outcome.

Prior use of thyroid hormones was consistently associated with lower risk of infection and severity, no matter whether the general population or test-negative individuals were considered as controls. The ORs were similar across all time windows. For model E (infected vs. population), the OR for 1-year time window was 0.80 (CI 0.71 to 0.92), which was close to the effect size under model F (infected vs. test-negative). For model C (hospitalized/fatal cases vs. population), the OR for 1-year time window was 0.62 (CI 0.48 to 0.79), and it was similar when constrained to tested subjects.

#### 3.1.5. Drugs Ranked by Consistency of Protective Associations

We also ranked the drugs in term of their *consistency* of protective associations. Briefly, drugs were ranked by their frequency of being at least nominally significant (*p* < 0.05) across the four time windows and eight models (Table 4). This serves as an alternative approach to prioritize drugs. For some drugs, the results may not be significant after FDR correction. Nevertheless, if a drug showed consistent associations (at least nominally) across multiple models or time-frames, it may also be worthy of further investigation.

#### 3.1.6. Drug Associated with Increased Odds of Risk/Severity of Infection

Among the drugs with harmful associations, the more frequently top-listed ones include laxatives, opioids (N02AA), benzodiazepines, tetracycline, penicillins, other antipsychotics (N05AX), and antidementia drugs (N06DA/DX). The full results are presented in Tables S6–S10, and a summary is also provided in Table S5.

### 3.2. Analysis Restricted to Subjects with Complete Covariate Data, and Models with/without IPW

As a sensitivity analysis, for the above analysis with imputed covariates, we also repeated models A to H *without* IPW of Pr(tested). In addition, we also repeated the analyses, limiting to subjects with complete covariate data, with or without the IPW approach. In general, we observed similar drugs with significant results, and the top-ranked protective or harmful drugs were similar to the above. Comparing results with and without IPW, the list of significant drugs remained similar although the OR estimates and SE were adjusted. The full results are presented in Tables S7 and S8 (complete covariate data with and without IPW) and Table S9 (imputed covariates without IPW).

### 3.3. Subgroup Analysis

The proportion of subjects falling into each subgroup is presented in Table S10, while full results are presented in Table S11. We performed a statistical test to compare the log(OR) across the two subgroups with and without the risk factor; drugs with protective effect in one subgroup but significantly different OR in the other subgroup are listed in Table 7. For example, the protective effects of pneumococcal and flu vaccines were significantly weaker in obese (BMI > 30) subjects under model F. With regards to age, several drugs, such as PPI and ACEI, showed larger protective effects in those with age > 70 under models F and E, respectively. Statins, ACEIs, and PPI showed stronger protective associations in hypertensive patients under models C, E, and F, respectively. Regarding ethnicity as a subgroup, a number of drugs, including several vaccines, appeared to have stronger protective effects in the white compared to non-white subjects. However, only <10% of the UKBB subjects included here were non-white, and the non-white subgroup was heterogeneous and composed of several different ethnicities. We did not observe clear evidence of sex-specific effects in this analysis.

### 3.4. Interaction Analysis

A summary of results (results with FDR < 0.2) is presented in Table 8, while a fuller version is given in Table S12. Full results are given in Table S13. More significant results (at FDR < 0.2) are observed compared to stratified analysis, presumably due to the higher power of this approach. For example, we found that most vaccines showing protective effects, including influenza and pneumococcal vaccines, interacted with BMI and obesity significantly. Higher BMI was associated with *reduced* protective effects, in line with evidence from subgroup analysis.

On the other hand, statins, biguanides (metformin), and antiplatelet drugs showed positive interactions with BMI. For CAD, significant interaction was observed with several cardiometabolic drugs, including beta-blockers (nonselective), antiplatelet drugs, and statins, suggesting larger protective effects for such drugs in CAD patients. In a similar vein, most cardiometabolic medications showed interaction with HT, indicating more prominent protective associations in HT patients.

Considering age as an interacting variable, interaction was observed with a large number of drugs, most suggesting weaker protection as age increases. Considering specific medications, statins interact with multiple risk factors and demonstrate larger protective effects with CAD, obesity, DM, CAD, HT, dementia, and in males. However, its effect tends to be weaker with increasing age. Interaction analysis with flu vaccines showed that its effect may be weaker in the obese and with increasing age, but was stronger in the white population and asthmatic subgroup. ACEI and ARB showed stronger protective effects in the white and HT patients, but weaker effects with advanced age.

### 3.5. Controlling for Other Medications

We primarily focused on protective drugs, as the number of drugs with significant negative effects is large and is hard to control for all. Overall, most drugs with protective effects remain significant (at least for a subset of models), despite controlling for other medications (Table S15). However, biguanides (A10BA), CCB (C08CA), and platelet aggregation inhibitors, excluding heparin (B01AC), showed a relatively consistent trend of nonsignificant association with outcome when other protective drugs were controlled for. The findings are similar when controlling for top-10/20 drugs or all protective drugs having FDR < 0.05/0.1.

## 4. Discussion

In this work, we performed a thorough and rigorous analysis on the effect of drugs and vaccines on COVID-19 susceptibility and severity. We uncovered a number of drugs with potentially protective effects, which may be further explored as candidates for drug repositioning.

As an approach based on observational data, different kinds of bias, such as confounding and selection bias, may affect the results. We performed analysis on infected subjects (models A and B), the whole population (models C, D, E) and the tested population (models F, G, H) to obtain a more comprehensive picture of drug effects under different settings, and to avoid limitations (e.g., selection bias, collider bias, unscreened controls) of some designs.

### 4.1. Highlights of Relevant Drugs

Below, we highlight drugs that are tentatively associated with altered risk or severity of infection. We preferentially consider drugs that showed significant associations across multiple models and time windows, those with stronger statistical significance, and those with protective effects, as confounding by indication is much less likely.

#### 4.1.1. Drugs for Cardiometabolic Disorders with Protective Effects

Interestingly, many drugs with potential protective effects are indicated for cardiometabolic (CM) disorders. Cardiometabolic risk factors, such as obesity, hypertension, DM, and CAD, have consistently been shown to be associated with risk and severity of infection [15,40]; as such, it is biologically plausible that drugs for treating CM disorders may be beneficial.

Among all drugs, the strongest and most consistent protective association was observed for statins. The beneficial effects of statins are supported by several previous studies. For example, a recent meta-analysis of four retrospective studies of COVID-19 patients [41] showed a significantly decreased hazard of severity or mortality of infection (pooled HR = 0.70) when comparing statin users against nonusers. Another retrospective study by Tan et al. [42] also reported lower risk of intensive care unit (ICU) admission among statin users in infected patients. Yet another work showed that statins may be effective in reducing in-hospital mortality among diabetic patients [43]. Potential mechanisms for the protective actions of statins have been discussed elsewhere [44,45,46]. It has been postulated that, besides reducing CVD risks, statins may reduce risk/severity of infection by inhibiting inflammation and excessive immune response, producing direct antiviral effects, improving endothelial function, and exerting an antithrombotic effect, among other actions [44,45,46].

Another group of drugs worth highlighting is ACEI and ARB. There have been intense discussions on whether ACEI/ARB may affect risk or severity of infection from early on, as ACE2 is a receptor for SARS-CoV-2. Nevertheless, a recent study showed that ACE2 is localized in respiratory cilia, and the use of ARB/ACEI does not change its expression [47]. Recent systemic reviews and meta-analysis (for example, see [48] with continuous updates) of observational studies do not support an association between ACEI/ARB prior use and severity of infection. However, several studies [47,49,50,51,52,53,54,55] reported protective effects of ACEI/ARB on severity or mortality of disease. Here, we observed consistent association of prior use of ACEI/ARB with reduced risks of severe/fatal infection (models A, C, G) and overall infection risk in the population (model E).

For several other kinds of cardiometabolic drugs, the associations were not as strong, but may still be worthy of further studies. Biguanides (mainly metformin) are observed to be protective for severe COVID-19 infection, both among the infected and at a population level. For example, in a meta-analysis on four observational studies of hospitalized patients mostly with type 2 DM, the use of metformin was associated with a lower risk of mortality (OR = 0.75, 95% CI = 0.67–0.85) [56]. A number of mechanisms have been proposed [56,57]. For example, besides improving glycemic control and weight reduction, metformin may lead to AMPK activation which potentially reduces viral entry by phosphorylation of ACE2 receptor. It may also lead to mTOR pathway inhibition and prevents hyperactivation of the immune system [56].

Other drugs of interest may include beta-blockers and calcium channel blockers (C08CA, dihydropyridine derivatives). It was suggested that beta-blockers may be useful in preventing hyperinflammation and hence beneficial for COVID-19 [58]. For calcium channel blockers (CCBs), a study using cell culture suggested that CCBs, especially amlodipine and nifedipine, were useful in blocking viral entry and infection in epithelial lung cells [59]. In another retrospective study [60], both beta-blockers and CCBs were associated with lower mortality. Another relevant study in the UK [61] utilized data from the UK Clinical Practice Research Datalink (CPRD) and found that ACEI/ARB, CCBs, and thiazide diuretics were all associated with lower odds of diagnosis, while beta-blockers do not show any association after adjusting for consultation frequency. None of the above drugs were associated with mortality in that study [61].

#### 4.1.2. Vaccines

There has been intense interest in whether vaccines indicated for other diseases may protect against COVID-19. Here, we observed that a number of vaccines showed protection against infection or severe infection. For example, pneumococcal vaccines were protective against infection in the population and tested subjects, and risk of severe infection (model G). Significant protective associations were also observed for tetanus and typhoid vaccines at a time horizon of 10 years (the power to detect associations is likely stronger over longer periods due to larger number of people having received the vaccine; it does not exclude the possibility that the vaccines may have effects over shorter time windows). We also observed associations with the J07CA category, which contains various bacterial and viral vaccines (see https://www.whocc.no/atc_ddd_index/?code=J07CA, accessed on 9 November 2020).

For influenza vaccines, we observed highly consistent protective associations. It has been proposed that “trained innate immunity”, which may involve epigenetic reprogramming of innate immune cells, may enable a vaccine to protect against other diseases [62,63]. Interestingly, two studies in Italy reported that higher coverage rate of flu vaccine was associated with lower rate of infection, hospitalization, and mortality from COVID-19. Another larger-scale study, based on electronic records of 137,037 subjects who have received viral PCR tests, showed that a number of vaccines (given in the past 1, 2, or 5 years) were associated with lower risks of infection [64]. These included flu and pneumococcal vaccines also implicated in the present study. Another recent study in the Netherlands [65] also showed a reduced risk (Relative risk = 0.61, 95% CI: 0.46–0.82) of infection among recipients of flu vaccine, and this effect size was similar to that observed here. In vitro studies by the same authors showed that the vaccine was able to induce a trained immunity response, including an increase of cytokine responses after stimulation of immune cells with SARS-CoV-2.

We note that this is an observational study, and residual confounding may be present. For example, it is possible that people receiving flu vaccines are more health-conscious and observe preventive measures better. However, we observed waning protective effects over time, which makes sense biologically but could not be entirely explained by the above confounder alone. In addition, the vaccine appears to have stronger effect sizes if fatal infection is considered as the outcome (although the confidence interval is large), which cannot be easily explained by health-consciousness. On the other hand, as flu vaccines are more likely to be received by the elderly and those with chronic illnesses, residual confounding of these factors tend to push the effects towards the harmful side.

Taken together, we believe that the protective effects of vaccines may not be easily and fully explained away by other confounders. Further experimental and clinical studies are warranted to investigate the nonspecific effects of flu and other vaccines, especially since COVID-19 vaccines may not be easily available to many people (especially those in low-income countries) in a short timeframe.

#### 4.1.3. Other Potential Protective Drugs

We briefly highlight a few other drugs with potential protective effects. Estrogens (G03CA) were among the drugs showing protective associations. As many studies reported higher risks of severe disease in men than in women, it has been hypothesized that estrogen may play a part in the sex-discordant outcomes, for example via its effects on immune response to infections [66,67,68].

Thyroid hormones (TH) were also among the top-ranked drugs. It was postulated that TH may ameliorate tissue injury due to hypoxia by suppression of p38 MAPK [69]. Clinical trials on TH are ongoing [69,70], and our findings support a protective role of TH in COVID-19.

Another drug category of note is proton pump inhibitors (PPI). Several studies have suggested harmful effects of PPI on disease severity, which may be related to reduced gastric acid production with subsequent bacterial overgrowth [71,72,73]. However, an in vitro screening study revealed that PPIs may serve as a potent inhibitor of SARS-CoV-2 replication [74]. The difference in findings between the current study and previous works may be due to heterogeneity in study samples and designs, differences in the outcome studied (e.g., hospitalization vs. ICU admission used in some other studies; infection risk vs. severity of disease, etc.), and variations in the covariates being adjusted for. Residual confounding, such as by other comorbidities and drugs given, may also affect the results. Interestingly, we observed that effects of PPI may be stronger in certain subgroups (e.g., older age, HT), which may also account for the discrepancy in results across different studies.

Several other top-ranked drug categories in Table 4 may also be worth discussing. Testosterone-5-alpha reductase inhibitors (5ARis) were recently shown in a small randomized controlled trial (RCT) to reduce the time to remission [75]. Two earlier observational studies also reported lower risk of ICU admission and frequency of symptoms [76,77]; 5ARis block the conversion of testosterone to its more potent form, dihydrotestosterone. Of note, one of the key receptors for the SAR-CoV-2 virus is TMPRSS2 [78], and the only known promoter of the gene is an androgen response element in the promoter region [79].

Another drug category of interest is platelet aggregation inhibitors (B01AC). It has been reported that COVID-19 is associated with higher risk of thrombotic events, including deep vein thrombosis and pulmonary embolism [80]. Antithrombotic therapies have been hypothesized to reduce thrombo-inflammatory processes as a result of endothelial dysfunction related to viral infection [81]. An observational study reported that aspirin is associated with reduced risk of mechanical ventilation and mortality in hospitalized patients [82]; however, RCTs are lacking.

For some of the protective drugs highlighted above, we note that their significance weakened (or became nonsignificant) when controlling for other medications. However, we expect multicollinearity among the drug variables, as cardiometabolic disorders are highly comorbid and one patient often takes multiple medications. Multicollinearity may render interpretation of individual predictors difficult due to unstable coefficient estimates [83].

In our secondary analyses, we also considered *subgroup and interaction effects*. While this is a more exploratory analysis and further replications are required, it shed light on how the effects of drugs/vaccines may differ in people with different clinical background and may contribute to more “personalized” drug repositioning in the future. For instance, we observed a consistent trend that the protective associations of flu and pneumococcal vaccines were weaker in obese individuals. As an example, comparing those who received flu vaccine in the past season (2019–2020) against those who did not, the estimated OR for infection was 0.76 in the obese group and 0.54 in the non-obese group (model F). It has been observed before that obese individuals respond less well to flu and other vaccines due to impaired immunological responses [84,85]. As another example, statins were observed to have more prominent protective effects in those with cardiometabolic abnormalities, such as DM, HT, CAD, and obesity. This is also supported by a recent study [43] which showed mortality reduction in statin users in diabetic patients only.

#### 4.1.4. Drugs with Potentially Harmful Effects

We noted a number of drugs with potentially harmful effects, but we caution that residual confounding, such as confounding by indication, other comorbidities, and general poor health, may lead to bias towards an increased odds of infection or severe disease.

For example, people who have poorer health in general may visit their GPs more often and be prescribed drugs (e.g., laxatives, antibiotics, painkillers), which may lead to confounding. Nevertheless, it is possible that some of the top-ranked drugs may indeed increase the risk/severity of infection. For instance, it is slightly unexpected that laxatives were highly significant across multiple models and time windows. It has recently been postulated that dysregulation of gut microbiome may be associated with susceptibility or resilience to infection [86,87], and laxatives represent a main category of drugs that affect the gut microbiome [88]. Interestingly, several associations involve psychiatric medications such as benzodiazepines, antipsychotics, and antidementia drugs. The association may be due to underlying neuropsychiatric conditions (e.g., anxiety, psychosis, dementia, etc.), or the effect of the drugs, or a combination of both. Some of the above drugs overlap with those revealed in a recent study using primary care data in Scotland. In a univariate analysis restricted to nonresidents in care homes and those without major conditions, laxatives, anxiolytics, penicillins, and opioid analgesics were significantly associated with ICU admission or mortality from COVID-19 when compared to population controls [89]. These drugs were also top-listed as drugs with harmful effects in this study.

Patients taking immunosuppressants are more susceptible to viral infections in general, and it is possible that these drugs are also associated with increased vulnerability to COVID-19 infection [90]. On the other hand, such drugs may dampen excessive immune responses (“cytokine storm”) that may occur in severe infections [91]. However, here we did not find consistent evidence of associations between immunosuppressive agents and COVID-19. Across immunosuppressive drugs (ATC category L04), we only found two significant associations (FDR < 0.05). Interleukin inhibitors were associated with higher susceptibility to infection (model E) and selective immunosuppressants (L04AA) were associated with higher risk of severe infection (model C), respectively, when compared to population controls (Table S6). No other significant associations were observed. Of note, a few preclinical studies reported that thiopurines, a type of immunosuppressant, may lead to reduced viral replication [92,93] via other mechanisms, although clinical studies suggested possible harmful effects [94,95]. However, the number of patients taking such drugs was too small for meaningful analysis in this study.

#### 4.1.5. Different Results under Different Models

We note that sometimes the different models may yield different results. One main observation is that analysis on the tested population appears to result in more findings of drugs with protective effects. We also observed that some drugs in model F (infected vs. tested negative) may show different effects under model E (infected vs. general population). Several reasons may explain this finding. First, confounding by indication is inevitable and may play a more important role when analyzing general population samples. It is possible that apparent harmful effects of drugs are due to the diseases/conditions that the prescription is related to, or poorer health in general. Based on a machine learning model for predicting testing probability (see Figure S1), we observed that people who are older, having more comorbidities and taking more medications, suffering from cardiovascular conditions, etc. were more likely to be tested. Compared to the general population, the tested group may represent a more “homogeneous” population, enriched for people with poorer health and more comorbidities in general. Therefore, a proportion of confounders which overlap with factors associated with higher Pr(tested) are essentially controlled for by stratification, if we only study the tested subjects. On the other hand, in the general population, as there is a higher proportion of healthy subjects, the effect of confounding by indication may be stronger. Another possibility is collider bias due to conditioning on a subgroup of subjects. For example, a drug may be associated with certain conditions which, in turn, are associated with higher chance of being tested; on the other hand, those who have more severe symptoms or complications are more likely to be tested. Conditioning on testing may result in spurious associations between the drug and severity of infection. However, we have tried to minimize this type of bias by the IPW approach, and we did not observe substantial difference in results with or without IPW correction for most drugs. However, we note that, even with adjustment by IPW, there is still chance for residual selection or collider bias. For example, some factors associated with Pr(tested) may not be captured in the prediction model. A third possibility to consider is that a drug may truly produce different effects in different subgroups, due to effect modification by other factors or diseases. For instance, a recent study reported that the protective effect of statins is more marked in patients with diabetes [43]. The fact that risk factor associations may differ between a whole-population- or tested-population-based study has also been noted previously, for example in [35].

### 4.2. Strengths and Limitation

This study has a number of strengths. First and foremost, the study was performed on a large cohort with a sample size close to half a million. The sample was not limited to one or a few medical centers, and covered the entire UK population, although this is not an entirely random sample and participation bias still exists [34]. The large and well-characterized sample also enables analysis of infected and tested, as well as the whole population. We have studied *all* level-4 ATC drug categories, allowing an unbiased and systematic analysis on the association of different drugs with COVID-19 risks or outcomes. This avoids the risk of publication bias, especially negative results to be unreported. Drugs showing null associations can still be of important public health interest, as this may suggest that patients on such medications may not need to change their regimen in view of the pandemic. In addition, medication history was retrieved from GP records, which minimize recall bias and errors from self-reporting. Another strength is that we performed a variety of statistical analysis to reduce bias, including control for potential confounders, multiple imputation, IPW to reduce effects of testing bias, and study of different time windows and multiple models. Some of our findings were also corroborated by previous studies. Many previous clinical studies were limited to hospitalized or infected individuals, which cannot study the effect of drugs on susceptibility to infection. Selection on hospitalized/infected subjects may also be prone to selection/collider bias, as discussed elsewhere [34]; therefore, we included multiple models with infected and tested, as well the whole population as samples, which aims to reduce limitations due to specific designs.

There are also various limitations, some of which have been mentioned above. First and foremost, this is an observational study based on a retrospective cohort of UKBB. As this is not a randomized controlled trial, confounding is inevitable, especially confounding by indication. Although we have controlled for main confounders in the regression model, residual confounding is still likely. Since confounding by indication will likely bias towards *increased* odds of infection or severe disease, null or protective associations may be more reliable. Confounding by the use of other types of drugs is also possible. In addition, the UKBB cohort is not random, and participants are on average healthier than the general population [96]. The majority of participants are of European descent, so the findings may not be generalizable to other ethnicities. In addition, the subjects are mostly >50 years old, and drug effects in younger individuals may be different.

Regarding drug history, it is worth noting that vaccination records are not complete, as individuals may receive vaccination outside GP practices. Over-the-counter prescriptions were not counted, and it cannot be guaranteed that all drugs issued are dispensed by the pharmacy (see https://biobank.ctsu.ox.ac.uk/crystal/crystal/docs/tppgp4covid19.pdf, accessed on 9 November 2020). However, if this misclassification is nondifferential (unrelated to outcome), the bias will be towards the null. There is a relatively high missing rate of GP prescription records for deceased COVID-19 patients, which leads to reduced power to detect associations. While the UKBB cohort sample is large, we still have low power to detect associations for drugs that are uncommonly prescribed. Another limitation with the GP records is that only the issue date, but no duration or dosage, is available.

As for the outcome, hospitalization is a rough proxy for severity only. For models comparing to the general population, it is likely that a proportion of the population may be infected but were not tested. This tends to lead to bias on the conservative side (akin to the use of unscreened controls in genetic studies [97,98]), especially under model E. Patients with more severe symptoms are less likely to remain untested, so other models may be less affected by this bias. We note that this study focuses on prior (or pre-diagnostic) use of drugs and their association with infection risk/severity, and does not provide direct evidence for whether newly prescribed drugs to recently diagnosed patients will be useful or not. The current study represents one approach to drug repositioning with real-world population data, yet integrating results from other repositioning approaches (e.g., network/structure-based) may further improve the reliability of candidates.

### 4.3. Clinical Implications

We highlight a few clinical implications here, although we stress that further studies are required to confirm our findings. We discovered a number of drugs with potential protective effects that, if replicated and tested in further trials, may represent promising repurposing candidates (for prevention or treatment of disease). As CM disorders are a major risk factor for severe infection, this study also provides further support for the safety of CM medications and reinforces the need to continue these drugs for those indicated. In a similar vein, negative findings (nonsignificant associations with COVID-19) in this study may also be of value, given that some patients or physicians may have concerns over the risk of COVID-19 induced by existing drugs.

Another important finding is that flu (and possibly others, e.g., pneumococcal) vaccines may be associated with lower odds of infection and severity of disease. If further confirmed, the finding is clinically important as COVID-19 vaccines are not fully available yet to a large part of the world’s population (especially those in developing countries), some may be hesitant to take the new vaccine, and the efficacy of existing vaccines varies and is less than perfect. At least, the present work supports that flu and other vaccinations should be continued and encouraged amid the pandemic. For any vaccines/drugs that may be repurposed for COVID-19, we believe that even a modest reduction in the risk/severity of infection may still be highly useful, given the huge number of people at risk for COVID-19 and its complications.

## 5. Conclusions

Here, we observed that a number of drugs, including many for cardiometabolic disorders, may be associated with lower odds of infection/severity of COVID-19. Several existing vaccines, especially flu vaccines, may be beneficial against COVID-19 as well. Due to the observational nature of the study, confounding cannot be excluded, and other limitations may be present. We understand that causal relationship between drugs and disease cannot be reliably concluded from this study alone, and shall regard the findings as more exploratory than confirmatory. Nevertheless, to our knowledge, this is the most comprehensive study to date on drug/vaccine associations with COVID-19. We believe that the current work provides a valuable resource to prioritize repositioning candidates for future meta-analyses, clinical trials, and/or experimental studies.

## Figures and Tables

**Table 1:** The eight sets of analyses based on infected patients (model A, B), tested subjects (models F, G, H) and the population (models C, D, E).

Model	Cohort 1	Cohort 2
A	Hospitalized or fatal infection (U07.1) (Severe)	Non-hospitalized COVID-19 (Mild)
B	U07.1 cases	All other COVID-19 cases
C	Hospitalized or fatal infection (U07.1) (Severe)	UKBB subjects without COVID-19 Dx or tested-ve
D	U07.1 cases	UKBB subjects without COVID-19 Dx or tested-ve
E	Infected	UKBB subjects without COVID-19 Dx or tested-ve
F	Infected	Tested-ve
G	Hospitalized or fatal infection (U07.1) (Severe)	Tested-ve
H	U07.1 cases	Tested-ve

U07.1 is the code for fatal (laboratory-confirmed) COVID-19 infection based on the latest ICD coding. Dx, diagnosis; -ve, negative.

**Table 2:** Number of available subjects for analysis for the 8 models.

Model	Cohort 1	Cohort 2	Total
A	1318	2540	3858
B	170	3688	3858
C	1318	393,142	394,460
D	170	393,142	393,312
E	3858	393,142	397,000
F	3858	26,977	30,835
G	1318	26,977	28,295
H	170	26,977	27,147

Only subjects with available GP prescription records are shown.

**Table 3:** Cardiometabolic medications showing significant protective associations (limited to FDR < 0.05) within time windows of 6, 12, and 24 months.

Window	Model	ATC Code	OR	conf.low	conf.high	*p*	FDR.BH	Full Name
1 year	C	A10BA	0.67	0.51	0.88	4.01 × 10^−3^	1.11 × 10^−2^	Biguanides
2 years	C	A10BA	0.68	0.52	0.90	5.79 × 10^−3^	1.68 × 10^−2^	Biguanides
0.5 year	F	C07AB	0.78	0.68	0.89	3.56 × 10^−4^	7.40 × 10^−3^	Beta blocking agents, selective
1 year	F	C07AB	0.80	0.70	0.91	7.59 × 10^−4^	1.29 × 10^−2^	Beta blocking agents, selective
2 years	F	C07AB	0.78	0.69	0.88	9.10 × 10^−5^	2.15 × 10^−3^	Beta blocking agents, selective
1 year	C	C08CA	0.76	0.64	0.90	1.31 × 10^−3^	4.23 × 10^−3^	Dihydropyridine derivatives
2 years	C	C08CA	0.78	0.66	0.92	3.27 × 10^−3^	1.11 × 10^−2^	Dihydropyridine derivatives
0.5 year	A	C09AA	0.68	0.53	0.87	2.11 × 10^−3^	1.43 × 10^−2^	ACE inhibitors, plain
0.5 year	C	C09AA	0.75	0.62	0.91	3.15 × 10^−3^	6.48 × 10^−3^	ACE inhibitors, plain
0.5 year	G	C09AA	0.68	0.56	0.83	1.13 × 10^−4^	1.54 × 10^−3^	ACE inhibitors, plain
1 year	A	C09AA	0.68	0.54	0.86	1.15 × 10^−3^	1.03 × 10^−2^	ACE inhibitors, plain
1 year	C	C09AA	0.61	0.51	0.74	1.59 × 10^−7^	1.25 × 10^−6^	ACE inhibitors, plain
1 year	D	C09AA	0.57	0.36	0.92	2.22 × 10^−2^	4.31 × 10^−2^	ACE inhibitors, plain
1 year	E	C09AA	0.79	0.72	0.88	1.40 × 10^−5^	8.63 × 10^−5^	ACE inhibitors, plain
1 year	G	C09AA	0.71	0.59	0.85	2.80 × 10^−4^	4.00 × 10^−3^	ACE inhibitors, plain
2 years	A	C09AA	0.67	0.54	0.84	5.87 × 10^−4^	1.10 × 10^−2^	ACE inhibitors, plain
2 years	C	C09AA	0.63	0.53	0.75	2.84 × 10^−7^	2.60 × 10^−6^	ACE inhibitors, plain
2 years	E	C09AA	0.81	0.73	0.90	5.38 × 10^−5^	3.41 × 10^−4^	ACE inhibitors, plain
2 years	G	C09AA	0.71	0.59	0.85	1.40 × 10^−4^	2.81 × 10^−3^	ACE inhibitors, plain
1 year	C	C09CA	0.68	0.54	0.85	7.58 × 10^−4^	2.61 × 10^−3^	Angiotensin II receptor blockers, plain
1 year	G	C09CA	0.69	0.55	0.87	1.95 × 10^−3^	1.85 × 10^−2^	Angiotensin II receptor blockers, plain
2 years	C	C09CA	0.73	0.58	0.90	3.97 × 10^−3^	1.25 × 10^−2^	Angiotensin II receptor blockers, plain
2 years	G	C09CA	0.72	0.58	0.90	3.93 × 10^−3^	4.80 × 10^−2^	Angiotensin II receptor blockers, plain
0.5 year	A	C10AA	0.57	0.47	0.68	3.37 × 10^−9^	8.37 × 10^−8^	HMG CoA reductase inhibitors
0.5 year	C	C10AA	0.79	0.68	0.91	1.20 × 10^−3^	2.63 × 10^−3^	HMG CoA reductase inhibitors
0.5 year	E	C10AA	1.14	1.05	1.24	1.64 × 10^−3^	4.26 × 10^−3^	HMG CoA reductase inhibitors
0.5 year	G	C10AA	0.66	0.57	0.76	2.55 × 10^−8^	9.03 × 10^−7^	HMG CoA reductase inhibitors
1 year	A	C10AA	0.50	0.42	0.60	2.87 × 10^−13^	5.17 × 10^−11^	HMG CoA reductase inhibitors
1 year	C	C10AA	0.49	0.42	0.57	2.97 × 10^−21^	7.42 × 10^−20^	HMG CoA reductase inhibitors
1 year	D	C10AA	0.50	0.34	0.74	5.28 × 10^−4^	1.57 × 10^−3^	HMG CoA reductase inhibitors
1 year	E	C10AA	0.83	0.77	0.91	1.69 × 10^−5^	1.00 × 10^−4^	HMG CoA reductase inhibitors
1 year	G	C10AA	0.63	0.54	0.73	4.15 × 10^−10^	2.77 × 10^−8^	HMG CoA reductase inhibitors
2 years	A	C10AA	0.49	0.40	0.58	1.55 × 10^−14^	3.19 × 10^−12^	HMG CoA reductase inhibitors
2 years	C	C10AA	0.49	0.43	0.57	7.09 × 10^−21^	2.60 × 10^−19^	HMG CoA reductase inhibitors
2 years	D	C10AA	0.50	0.34	0.74	4.38 × 10^−4^	1.63 × 10^−3^	HMG CoA reductase inhibitors
2 years	E	C10AA	0.86	0.79	0.93	3.09 × 10^−4^	1.52 × 10^−3^	HMG CoA reductase inhibitors
2 years	G	C10AA	0.63	0.54	0.72	2.65 × 10^−10^	2.92 × 10^−8^	HMG CoA reductase inhibitors

For space limits, only results with FDR < 0.05 are shown. Please refer to Tables S3 and S6 for full results. OR, odds ratio; conf.low, lower 95% CI for OR; conf.high, upper 95% CI for OR; FDR.BH, false discovery rate by the Benjamini–Hochberg method.

**Table 4:** Drugs showing consistent protective associations across 4 time-windows and 8 models (ranked by the frequency of being nominally significant, i.e., *p* < 0.05).

	ATC Code	Drug Name	Freq
1	C09AA	ACE inhibitors, plain	21
2	J07BB	Influenza vaccines	20
3	C10AA	HMG CoA reductase inhibitors	19
4	H03AA	Thyroid hormones	17
5	C09CA	Angiotensin II receptor blockers, plain	15
6	G04CB	Testosterone-5-alpha reductase inhibitors	12
7	A02BC	Proton pump inhibitors	11
8	C08CA	Dihydropyridine derivatives	11
9	R03BA	Glucocorticoids	9
10	C07AB	Beta blocking agents, selective	8
11	A10BA	Biguanides	7
12	B01AC	Platelet aggregation inhibitors excl. heparin	7
13	G03CA	Natural and semisynthetic estrogens, plain	7
14	J07CA	Bacterial and viral vaccines, combined	7
15	A03AA	Synthetic anticholinergics, esters with tertiary amino group	6

Frequency (freq) calculated based on results from time windows of 6 months to 5 years. Ophthalmological and dermatological agents are not listed in the above table.

**Table 5:** Vaccines with significant protective associations (limited to FDR < 0.05) within time windows of 1, 2, 5, and 10 years.

Window	Model	ATC Code	OR	conf.low	conf.high	*p*	FDR.BH	Full Name
1 year	F	J07AL	0.50	0.31	0.82	5.29 × 10^−3^	4.65 × 10^−2^	Pneumococcal vaccines
2 years	F	J07AL	0.59	0.42	0.82	1.59 × 10^−3^	2.17 × 10^−2^	Pneumococcal vaccines
5 years	E	J07AL	0.70	0.55	0.89	3.81 × 10^−3^	1.62 × 10^−2^	Pneumococcal vaccines
5 years	F	J07AL	0.61	0.47	0.79	1.47 × 10^−4^	3.27 × 10^−3^	Pneumococcal vaccines
10 years	E	J07AL	0.78	0.67	0.91	1.89 × 10^−3^	8.52 × 10^−3^	Pneumococcal vaccines
10 years	F	J07AL	0.67	0.57	0.78	9.39 × 10^−7^	4.23 × 10^−6^	Pneumococcal vaccines
10 years	G	J07AL	0.67	0.51	0.87	3.32 × 10^−3^	9.20 × 10^−3^	Pneumococcal vaccines
5 years	F	J07AM	0.45	0.29	0.68	1.93 × 10^−4^	3.73 × 10^−3^	Tetanus vaccines
10 years	E	J07AM	0.65	0.45	0.92	1.60 × 10^−2^	4.23 × 10^−2^	Tetanus vaccines
10 years	F	J07AM	0.49	0.34	0.71	1.69 × 10^−4^	3.80 × 10^−4^	Tetanus vaccines
5 years	F	J07AP	0.70	0.58	0.84	1.60 × 10^−4^	3.30 × 10^−3^	Typhoid vaccines
10 years	E	J07AP	0.86	0.76	0.97	1.88 × 10^−2^	4.23 × 10^−2^	Typhoid vaccines
10 years	F	J07AP	0.76	0.67	0.88	1.18 × 10^−4^	3.55 × 10^−4^	Typhoid vaccines
10 years	G	J07AP	0.74	0.58	0.95	1.61 × 10^−2^	2.82 × 10^−2^	Typhoid vaccines
1 year	C	J07BB	0.74	0.60	0.91	3.80 × 10^−3^	1.08 × 10^−2^	Influenza vaccines
1 year	D	J07BB	0.28	0.13	0.63	1.92 × 10^−3^	4.68 × 10^−3^	Influenza vaccines
1 year	E	J07BB	0.73	0.65	0.83	5.93 × 10^−7^	4.50 × 10^−6^	Influenza vaccines
1 year	F	J07BB	0.60	0.53	0.68	2.94 × 10^−15^	6.97 × 10^−13^	Influenza vaccines
1 year	G	J07BB	0.61	0.50	0.76	4.35 × 10^−6^	1.09 × 10^−4^	Influenza vaccines
1 year	H	J07BB	0.23	0.11	0.52	4.04 × 10^−4^	3.32 × 10^−3^	Influenza vaccines
2 years	C	J07BB	0.75	0.62	0.90	2.01 × 10^−3^	7.27 × 10^−3^	Influenza vaccines
2 years	D	J07BB	0.30	0.15	0.60	7.22 × 10^−4^	2.30 × 10^−3^	Influenza vaccines
2 years	E	J07BB	0.75	0.68	0.84	4.83 × 10^−7^	4.83 × 10^−6^	Influenza vaccines
2 years	F	J07BB	0.62	0.55	0.70	4.38 × 10^−16^	1.14 × 10^−13^	Influenza vaccines
2 years	G	J07BB	0.62	0.52	0.75	8.86 × 10^−7^	2.78 × 10^−5^	Influenza vaccines
2 years	H	J07BB	0.25	0.12	0.50	9.64 × 10^−5^	9.11 × 10^−4^	Influenza vaccines
5 years	D	J07BB	0.53	0.32	0.86	9.80 × 10^−3^	3.83 × 10^−2^	Influenza vaccines
5 years	E	J07BB	0.80	0.73	0.88	7.01 × 10^−6^	5.79 × 10^−5^	Influenza vaccines
5 years	F	J07BB	0.66	0.60	0.73	7.67 × 10^−16^	1.11 × 10^−13^	Influenza vaccines
5 years	G	J07BB	0.69	0.59	0.81	8.14 × 10^−6^	2.93 × 10^−4^	Influenza vaccines
5 years	H	J07BB	0.44	0.27	0.72	1.12 × 10^−3^	1.07 × 10^−2^	Influenza vaccines
10 years	D	J07BB	0.59	0.39	0.90	1.51 × 10^−2^	4.54 × 10^−2^	Influenza vaccines
10 years	E	J07BB	0.82	0.75	0.89	6.70 × 10^−6^	6.03 × 10^−5^	Influenza vaccines
10 years	F	J07BB	0.67	0.61	0.74	5.16 × 10^−17^	4.64 × 10^−16^	Influenza vaccines
10 years	G	J07BB	0.69	0.59	0.80	9.82 × 10^−7^	6.87 × 10^−6^	Influenza vaccines
10 years	H	J07BB	0.50	0.32	0.76	1.44 × 10^−3^	4.31 × 10^−3^	Influenza vaccines
1 year	F	J07CA	0.56	0.38	0.84	4.30 × 10^−3^	3.97 × 10^−2^	Bacterial and viral vaccines, combined
2 years	F	J07CA	0.71	0.57	0.89	3.05 × 10^−3^	3.59 × 10^−2^	Bacterial and viral vaccines, combined
10 years	F	J07CA	0.85	0.78	0.94	7.85 × 10^−4^	1.41 × 10^−3^	Bacterial and viral vaccines, combined
10 years	G	J07CA	0.78	0.66	0.92	3.94 × 10^−3^	9.20 × 10^−3^	Bacterial and viral vaccines, combined

For space limits, only results with FDR < 0.05 are shown. Please refer to Table S4 for full results.

**Table 6:** Other drugs with significant protective associations (limited to FDR < 0.05) within time windows of 6, 12, and 24 months.

Window	Model	ATC Code	OR	conf.low	conf.high	*p*	FDR.BH	Full Name
0.5 year	F	A02BC	0.72	0.67	0.79	1.05 × 10^−13^	2.18 × 10^−11^	Proton pump inhibitors
0.5 year	G	A02BC	0.70	0.61	0.81	1.06 × 10^−6^	2.08 × 10^−5^	Proton pump inhibitors
1 year	A	A02BC	0.77	0.65	0.91	2.37 × 10^−3^	1.78 × 10^−2^	Proton pump inhibitors
1 year	F	A02BC	0.77	0.71	0.83	2.01 × 10^−11^	2.38 × 10^−9^	Proton pump inhibitors
1 year	G	A02BC	0.66	0.58	0.76	1.56 × 10^−9^	7.80 × 10^−8^	Proton pump inhibitors
2 years	A	A02BC	0.77	0.66	0.90	1.05 × 10^−3^	1.80 × 10^−2^	Proton pump inhibitors
2 years	F	A02BC	0.80	0.74	0.86	2.94 × 10^−9^	2.55 × 10^−7^	Proton pump inhibitors
2 years	G	A02BC	0.68	0.59	0.77	1.81 × 10^−9^	9.96 × 10^−8^	Proton pump inhibitors
2 years	F	A03FA	0.51	0.37	0.70	3.67 × 10^−5^	1.19 × 10^−3^	Propulsives
1 year	F	A09AA	0.24	0.09	0.64	4.19 × 10^−3^	3.97 × 10^−2^	Enzyme preparations
2 years	F	A09AA	0.23	0.09	0.60	2.81 × 10^−3^	3.48 × 10^−2^	Enzyme preparations
0.5 year	F	A12AX	0.80	0.69	0.93	2.74 × 10^−3^	3.49 × 10^−2^	Calcium, combinations with vitamin D and/or other drugs
1 year	F	A12AX	0.83	0.72	0.94	4.36 × 10^−3^	3.97 × 10^−2^	Calcium, combinations with vitamin D and/or other drugs
1 year	F	B03AA	0.74	0.60	0.91	4.00 × 10^−3^	3.97 × 10^−2^	Iron bivalent, oral preparations
2 years	F	C05AE	0.33	0.16	0.69	3.18 × 10^−3^	3.59 × 10^−2^	Muscle relaxants
0.5 year	F	G03CA	0.63	0.52	0.76	3.03 × 10^−6^	1.58 × 10^−4^	Natural and semisynthetic estrogens, plain
1 year	F	G03CA	0.67	0.58	0.78	4.08 × 10^−7^	2.42 × 10^−5^	Natural and semisynthetic estrogens, plain
2 years	F	G03CA	0.70	0.61	0.80	1.89 × 10^−7^	9.83 × 10^−6^	Natural and semisynthetic estrogens, plain
2 years	G	G03CA	0.66	0.51	0.86	2.43 × 10^−3^	3.35 × 10^−2^	Natural and semisynthetic estrogens, plain
0.5 year	F	G04CB	0.63	0.46	0.85	3.02 × 10^−3^	3.49 × 10^−2^	Testosterone-5-alpha reductase inhibitors
0.5 year	F	H03AA	0.80	0.69	0.92	2.24 × 10^−3^	3.11 × 10^−2^	Thyroid hormones
0.5 year	G	H03AA	0.66	0.51	0.86	2.10 × 10^−3^	1.96 × 10^−2^	Thyroid hormones
1 year	C	H03AA	0.62	0.48	0.79	1.77 × 10^−4^	6.57 × 10^−4^	Thyroid hormones
1 year	E	H03AA	0.80	0.71	0.92	9.47 × 10^−4^	4.23 × 10^−3^	Thyroid hormones
1 year	F	H03AA	0.81	0.71	0.93	2.51 × 10^−3^	2.98 × 10^−2^	Thyroid hormones
1 year	G	H03AA	0.64	0.49	0.82	5.53 × 10^−4^	6.50 × 10^−3^	Thyroid hormones
2 years	C	H03AA	0.62	0.48	0.79	1.50 × 10^−4^	7.36 × 10^−4^	Thyroid hormones
2 years	E	H03AA	0.80	0.70	0.91	5.94 × 10^−4^	2.81 × 10^−3^	Thyroid hormones
2 years	F	H03AA	0.81	0.71	0.93	2.57 × 10^−3^	3.35 × 10^−2^	Thyroid hormones
2 years	G	H03AA	0.64	0.50	0.83	6.06 × 10^−4^	9.52 × 10^−3^	Thyroid hormones
1 year	F	J01MA	0.49	0.34	0.72	2.40 × 10^−4^	5.93 × 10^−3^	Fluoroquinolones
2 years	F	J01MA	0.59	0.46	0.76	5.39 × 10^−5^	1.56 × 10^−3^	Fluoroquinolones
0.5 year	F	L02AE	0.29	0.14	0.60	9.84 × 10^−4^	1.86 × 10^−2^	Gonadotropin releasing hormone analogues
1 year	F	L02AE	0.41	0.23	0.72	2.02 × 10^−3^	2.62 × 10^−2^	Gonadotropin releasing hormone analogues
2 years	F	L02AE	0.42	0.25	0.70	9.73 × 10^−4^	1.49 × 10^−2^	Gonadotropin releasing hormone analogues
0.5 year	F	M01AE	0.68	0.56	0.82	4.61 × 10^−5^	1.37 × 10^−3^	Propionic acid derivatives
1 year	F	M01AE	0.79	0.70	0.91	6.65 × 10^−4^	1.29 × 10^−2^	Propionic acid derivatives
0.5 year	F	N02AX	0.56	0.41	0.76	1.88 × 10^−4^	4.33 × 10^−3^	Other opioids
1 year	F	N02AX	0.63	0.49	0.80	1.63 × 10^−4^	4.84 × 10^−3^	Other opioids
2 years	F	N02AX	0.68	0.56	0.83	1.14 × 10^−4^	2.29 × 10^−3^	Other opioids
0.5 year	F	N03AX	0.68	0.58	0.81	1.72 × 10^−5^	5.96 × 10^−4^	Other antiepileptics
1 year	F	N03AX	0.70	0.60	0.82	1.00 × 10^−5^	3.95 × 10^−4^	Other antiepileptics
2 years	F	N03AX	0.73	0.64	0.84	7.15 × 10^−6^	3.10 × 10^−4^	Other antiepileptics
0.5 year	F	N06AA	0.77	0.65	0.92	3.99 × 10^−3^	4.15 × 10^−2^	Nonselective monoamine reuptake inhibitors
1 year	F	N06AA	0.79	0.68	0.92	1.98 × 10^−3^	2.62 × 10^−2^	Nonselective monoamine reuptake inhibitors
2 years	F	N06AA	0.79	0.70	0.90	2.67 × 10^−4^	4.96 × 10^−3^	Nonselective monoamine reuptake inhibitors
1 year	A	R03BA	0.48	0.31	0.73	7.44 × 10^−4^	7.44 × 10^−3^	Glucocorticoids
2 years	A	R03BA	0.55	0.38	0.81	2.44 × 10^−3^	3.36 × 10^−2^	Glucocorticoids
0.5 year	F	R05DA	0.69	0.55	0.87	1.46 × 10^−3^	2.33 × 10^−2^	Opium alkaloids and derivatives
1 year	F	R05DA	0.74	0.62	0.88	5.47 × 10^−4^	1.18 × 10^−2^	Opium alkaloids and derivatives
2 years	F	R05DA	0.80	0.70	0.91	7.02 × 10^−4^	1.22 × 10^−2^	Opium alkaloids and derivatives

For space limits, only results with FDR < 0.05 are shown. Please refer to Tables S3 and S6 for full results. Ophthalmological and other topical agents are not listed in the above table.

**Table 7:** Summary of subgroup analysis, showing drugs having significant protective association in one subgroup but significantly different OR in the other subgroup (FDR < 0.2).

Subgp	Windows	Model	OR_Y	OR_N	sig_Y	sig_N	z_OR_cmp	p_OR_cmp	p.adjust_OR_cmp	Name
AGE > 70	5 years	F	0.81	0.99	1	0	−2.65	8.15 × 10^−3^	1.47 × 10^−1^	A02BC Proton pump inhibitors
AGE > 70	1 year	E	1.19	0.49	0	1	2.11	3.47 × 10^−2^	1.56 × 10^−1^	A10AE Insulins and analogues for injection, long-acting
AGE > 70	1 year	E	0.81	1.04	1	0	−2.38	1.72 × 10^−2^	1.55 × 10^−1^	C09AA ACE inhibitors, plain
Asthma	5 years	E	0.60	0.86	1	1	−2.66	7.76 × 10^−3^	1.40 × 10^−1^	J07BB Influenza vaccines
Asthma	10 years	E	0.61	0.87	1	1	−2.95	3.22 × 10^−3^	1.29 × 10^−2^	J07BB Influenza vaccines
BMI > 30	1 year	F	1.04	0.31	0	1	2.42	1.56 × 10^−2^	1.40 × 10^−1^	J07AL Pneumococcal vaccines
BMI > 30	1 year	F	0.76	0.54	1	1	2.52	1.17 × 10^−2^	1.40 × 10^−1^	J07BB Influenza vaccines
BMI > 30	2 years	F	0.79	0.56	1	1	2.75	6.01 × 10^−3^	1.08 × 10^−1^	J07BB Influenza vaccines
BMI > 30	6 months	F	0.92	0.16	0	1	2.68	7.40 × 10^−3^	1.26 × 10^−1^	R03BA Glucocorticoids
CAD	5 years	H	0.36	1.32	1	0	−2.39	1.71 × 10^−2^	1.53 × 10^−1^	C08CA Dihydropyridine derivatives
CAD	5 years	H	1.72	0.18	0	1	2.42	1.53 × 10^−2^	1.53 × 10^−1^	G04CB Testosterone-5-alpha reductase inhibitors
CAD	5 years	C	1.92	0.56	0	1	2.38	1.72 × 10^−2^	1.55 × 10^−1^	J07AL Pneumococcal vaccines
CAD	5 years	F	1.55	0.56	0	1	2.55	1.07 × 10^−2^	1.92 × 10^−1^	J07AL Pneumococcal vaccines
Depression	1 yearr	B	0.07	0.73	1	0	−2.60	9.36 × 10^−3^	1.50 × 10^−1^	C10AA HMG CoA reductase inhibitors
HT	2 years	F	0.75	0.93	1	0	−2.69	7.20 × 10^−3^	1.30 × 10^−1^	A02BC Proton pump inhibitors
HT	5 years	F	0.76	1.00	1	0	−3.36	7.92 × 10^−4^	1.43 × 10^−2^	A02BC Proton pump inhibitors
HT	1 year	E	0.86	1.07	1	0	−2.11	3.49 × 10^−2^	1.26 × 10^−1^	C09AA ACE inhibitors, plain
HT	1 year	C	0.71	1.02	1	0	−2.58	9.90 × 10^−3^	8.91 × 10^−2^	C10AA HMG CoA reductase inhibitors

OR_Y, odds ratio within the subgroup defined in the 1st column; OR_N, OR in the other subgroup. Sig_Y, sig_N, significance in the two subgroups, 1 denotes significant protective effect, 0 denotes nonsignificant effect, −1 denotes significant harmful effect. p_OR_cmp, *p*-value based on comparison of ORs; p.adjust_OR_cmp, corresponding FDR. Ethnicity as a subgroup is not shown here; please refer to Table S11 for details. CAD, coronary artery disease, HT, hypertension.

**Table 8:** Summary of interaction analysis, showing pairs of variables with significant interactions (FDR < 0.2).

ATC Code	Interacting Factor	Drug Name	Interaction Term	ATC Code	Interacting Factor	Drug Name	Interaction Term
A02BC	AGE	Proton pump inhibitors	1/−1	A02BC	CAD	Proton pump inhibitors	1
A03AA	AGE	Synthetic anticholinergics, esters with tertiary amino group	−1	A03AA	CAD	Synthetic anticholinergics, esters with tertiary amino group	1
A10AE	AGE	Insulins and analogues for injection, long-acting	−1	B01AC	CAD	Platelet aggregation inhibitors excl. heparin	1
B01AC	AGE	Platelet aggregation inhibitors excl. heparin	−1	C07AB	CAD	Beta blocking agents, selective	1
C07AB	AGE	Beta blocking agents, selective	−1	C10AA	CAD	HMG CoA reductase inhibitors	1
C08CA	AGE	Dihydropyridine derivatives	−1	J07AL	CAD	Pneumococcal vaccines	−1
C09AA	AGE	ACE inhibitors, plain	−1	C10AA	Dementia	HMG CoA reductase inhibitors	1
C09CA	AGE	Angiotensin II receptor blockers, plain	−1	J07CA	Dementia	Bacterial and viral vaccines, combined	−1
C10AA	AGE	HMG CoA reductase inhibitors	−1	C08CA	COPD	Dihydropyridine derivatives	−1
G04CB	AGE	Testosterone-5-alpha reductase inhibitors	−1	J07AP	COPD	Typhoid vaccines	−1
J07AL	AGE	Pneumococcal vaccines	−1	A03AA	Depression	Synthetic anticholinergics, esters with tertiary amino group	−1
R03BA	AGE	Glucocorticoids	1	C10AA	DM	HMG CoA reductase inhibitors	1
A02BC	AGE > 70	Proton pump inhibitors	−1	A02BC	Dx_cancer	Proton pump inhibitors	−1
A10AE	AGE > 70	Insulins and analogues for injection, long-acting	−1	J07AL	Dx_cancer	Pneumococcal vaccines	−1
A10BA	AGE > 70	Biguanides	−1	A10BA	Ethnic (White)	Biguanides	1
B01AC	AGE > 70	Platelet aggregation inhibitors excl. heparin	−1	C08CA	Ethnic (White)	Dihydropyridine derivatives	1
C07AB	AGE > 70	Beta blocking agents, selective	−1	C09AA	Ethnic (White)	ACE inhibitors, plain	1
C08CA	AGE > 70	Dihydropyridine derivatives	−1	C09CA	Ethnic (White)	Angiotensin II receptor blockers, plain	1
C09AA	AGE > 70	ACE inhibitors, plain	−1	H03AA	Ethnic (White)	Thyroid hormones	1
C10AA	AGE > 70	HMG CoA reductase inhibitors	−1	J07AL	Ethnic (White)	Pneumococcal vaccines	1
G04CB	AGE > 70	Testosterone-5-alpha reductase inhibitors	−1	J07AP	Ethnic (White)	Typhoid vaccines	1
J07AL	AGE > 70	Pneumococcal vaccines	−1	J07BB	Ethnic (White)	Influenza vaccines	1
J07BB	AGE > 70	Influenza vaccines	−1	A02BC	Hypertension	Proton pump inhibitors	−1
R03BA	AGE > 70	Glucocorticoids	−1	A03AA	Hypertension	Synthetic anticholinergics, esters with tertiary amino group	1
A10AE	Asthma	Insulins and analogues for injection, long-acting	−1	B01AC	Hypertension	Platelet aggregation inhibitors excl. heparin	1
A10BA	Asthma	Biguanides	−1	C07AB	Hypertension	Beta blocking agents, selective	1
C08CA	Asthma	Dihydropyridine derivatives	−1	C08CA	Hypertension	Dihydropyridine derivatives	1
C09CA	Asthma	Angiotensin II receptor blockers, plain	−1	C09AA	Hypertension	ACE inhibitors, plain	1
J07AL	Asthma	Pneumococcal vaccines	−1	C09CA	Hypertension	Angiotensin II receptor blockers, plain	1
J07BB	Asthma	Influenza vaccines	1	C10AA	Hypertension	HMG CoA reductase inhibitors	1
A02BC	BMI	Proton pump inhibitors	1	J07AL	Hypertension	Pneumococcal vaccines	−1
A03AA	BMI	Synthetic anticholinergics, esters with tertiary amino group	−1	B01AC	Obesity	Platelet aggregation inhibitors excl. heparin	1
A10BA	BMI	Biguanides	1	C10AA	Obesity	HMG CoA reductase inhibitors	1
B01AC	BMI	Platelet aggregation inhibitors excl. heparin	1	J07AL	Obesity	Pneumococcal vaccines	−1
C10AA	BMI	HMG CoA reductase inhibitors	1	J07BB	Obesity	Influenza vaccines	−1
J07AL	BMI	Pneumococcal vaccines	−1	A02BC	Sex (male)	Proton pump inhibitors	1
J07AP	BMI	Typhoid vaccines	−1	C10AA	Sex (male)	HMG CoA reductase inhibitors	1
J07BB	BMI	Influenza vaccines	−1	J07AL	Sex (male)	Pneumococcal vaccines	−1
J07CA	BMI	Bacterial and viral vaccines, combined	−1	J07AP	Sex (male)	Typhoid vaccines	1

We added an interaction term drug*interacting factor in the regression model. For “interaction term”, 1 denotes significant interaction effects towards protection (i.e., presence of the interacting factor tends to increase the protective effect of the drug); −1 denotes significant interaction effects towards harmful side (presence of the interacting factor tends to reduce the protective effect of the drug). We consider significant results in any model or time window. For age and BMI, they were modeled as continuous variables unless otherwise specified. For full results, please refer to Tables S12 and S13.

## Data Availability

UK Biobank data are available to eligible researchers after completing an application procedure (https://www.ukbiobank.ac.uk/enable-your-research).

## Supplementary Materials

The following are available online at https://www.mdpi.com/article/10.3390/pharmaceutics13091514/s1, All supplementary Tables and notes are available at the journal’s website and at https://drive.google.com/drive/folders/1_noITkBAsef_7Kb6bUd_RI_3VQK5jafH?usp=sharing (accessed on 28 January 2021) or https://doi.org/10.6084/m9.figshare.14828112 (accessed on 23 June 2021). Table S1: Demographic and other characteristics of the original UKBB data, Table S2: Out-of-bag (OOB) errors for different variables from multiple imputations, Table S3: (a) All protective associations with at least nominal significance (*p* < 0.05) (6 month to 5 years), Table S4: All association results with vaccines (time windows of 1, 2, 5 and 10 years), Table S5: (a) Top 10 drugs with harmful effects (ranked by *p*-value) from each model and time window (time window of 6 month to 5 years) (b) Summary table by frequency of being listed among the top 10, Table S6: All association results based on subjects with available GP prescription records, with multiple imputation of covariates and inverse probability weighting (IPW) of probability of being tested, Table S7: Analysis restricted to subjects with complete covariate data, with IPW, Table S8: Analysis restricted to subjects with complete covariate data, without IPW, Table S9: Analysis with imputed covariates without IPW, Table S10: Proportion of subjects in each subgroup, Table S11: Full results of subgroup analysis, Table S12: Summary table of interaction analyses (results with FDR < 0.2), Table S13: Full results of interaction analyses, Table S14: Further analysis on associations of flu vaccine and risks/severity of infection, according to the season of vaccination, Table S15: Results of analyses after controlling for other top medications. Figure S1: Shapley dependence plot of top 15 variables contributing to Pr(tested) from the XGboost prediction model. Variables are ranked by absolute mean Shapley value. Please refer to Lundberg et al. (https://doi.org/10.1038/s42256-019-0138-9) for details on Shapley values.
