# Supplementary material for: Exploring Drugs and Vaccines Associated with Altered Risks and Severity of COVID-19: A UK Biobank Cohort Study of All ATC Level-4 Drug Categories Reveals Repositioning Opportunities
Source: Pharmaceutics. 2021 Sep 18;13(9):1514. doi: 10.3390/pharmaceutics13091514 (PMC8471264; doi:10.3390/pharmaceutics13091514)
Supplement: Supplementary file 1 [file pharmaceutics-13-01514-s001.zip › Summary of supplementary information.pdf]

Summary of supplementary information

All supplementary Tables and notes are available at the journal's website and at https://drive.google.com/drive/folders/1_noITkBAsef_7Kb6bUd_RI_3VQK5jafH?usp=sharing

Table S1 Demographic and other characteristics of the original UKBB data

Table S2 Out-of-bag (OOB) errors for different variables from multiple imputations

Table S3 a) All protective associations with at least nominal significance ($p<0.05$) (6 month to 5 years)

Legend:

OR: odds ratio; case_notx, number of subjects with *no* history of receiving the drug and belonging to 'case' (i.e. cohort 1 listed in main Table 1); case_tx, number of subjects with history of receiving the drug and belonging to 'case' (i.e. cohort 1 listed in main Table 1) ; ctrl_notx, number of subjects with *no* history of receiving the drug and belonging to 'control' (i.e. cohort 2 listed in main Table 1); ctrl_tx number of subjects with history of receiving the drug and belonging to 'control' (i.e. cohort 2 listed in main Table 1).

Estimate, estimated regression coefficient; std.error, standard error of coefficient; conf.low, lower 95% confidence interval of OR; conf.high, upper 95% confidence interval of OR; FDR.BH, false discovery rate by the Benjamini-Hochberg approach.

Sig: 1 denotes significant protective effect, 0 denotes non-significant effect, -1 denotes significant harmful effect.

P.adj: corresponding FDR.

b) Summary table by frequency of being nominally significant ($p<0.05$)

Table S4 All association results with vaccines (time-windows of 1, 2, 5 and 10 years)

Table S5 a) Top 10 drugs with harmful effects (ranked by p-value) from each model and time window (time window of 6 month to 5 years)

b) Summary table by frequency of being listed among the top 10

Table S6 All association results based on subjects with available GP prescription records, with multiple imputation of covariates and inverse probability weighting (IPW) of probability of being tested

Table S7 Analysis restricted to subjects with complete covariate data, with IPW

Table S8 Analysis restricted to subjects with complete covariate data, without IPW

Table S9 Analysis with imputed covariates *without* IPW

Table S10 Proportion of subjects in each subgroup

Table S11 Full results of subgroup analysis

p6m, past 6 months; p1yr, past 1 year (similar for other time windows); OR_Y, odds ratio within the subgroup

defined in the 1st column; OR_N, OR in the other subgroup. Sig_Y, sig_N, significance in the two subgroups, 1 denotes significant protective effect, 0 denotes non-significant effect, -1 denotes significant harmful effect. p_OR_cmp, p-value from comparison of two ORs in the two subgroups; p.adjust_OR_cmp, corresponding FDR. sig_OR_cmp, 1 denotes OR in the subgroup being less than the OR outside the subgroup, and vice versa.

Table S12 Summary table of interaction analyses (FDR<0.2)

We added an interaction term drug*interacting_factor in the regression model.

For 'interaction term', 1 denotes significant interaction effects towards protection (i.e. presence of the interacting factor tends to increase the protective effect of the drug); -1 denotes significant interaction effects towards harmful side (presence of the interacting factor tends to reduce the protective effect of the drug)

For age and BMI, they are modeled as continuous variables unless otherwise specified.

Table S13 Full results of interaction analyses

Legend: The coefficients, standard errors and p-values of main and interaction terms were shown. *X:Y* denotes the interaction term of *X**Y*.*

Table S14 Further analysis on effect flu vaccine and risks/severity of infection, according to the season of vaccination

Table S15 Results of analyses after controlling for other top medications

Legend: Headers ended with 1 (e.g. beta1, se1, p1 etc.) denote the original (unadjusted for other top medications) estimates while headers ended with 2 denote the estimates adjusted for other top medications.

Figure S1 Shapley dependence plot of top 15 variables contributing to Pr (tested) from XGboost model. Variables are ranked by absolute mean Shapley value. Please refer to Lundberg et al. (https://doi.org/10.1038/s42256-019-0138-9) for details on Shapley values.
