# Supplementary figures and images for: Exploring Drugs and Vaccines Associated with Altered Risks and Severity of COVID-19: A UK Biobank Cohort Study of All ATC Level-4 Drug Categories Reveals Repositioning Opportunities
Source: Pharmaceutics. 2021 Sep 18;13(9):1514. doi: 10.3390/pharmaceutics13091514 (PMC8471264; doi:10.3390/pharmaceutics13091514)

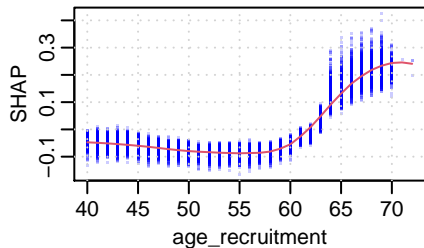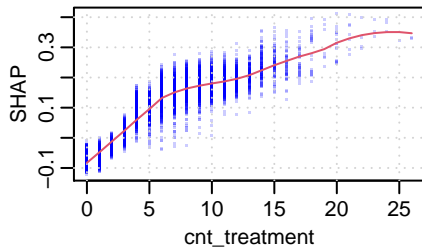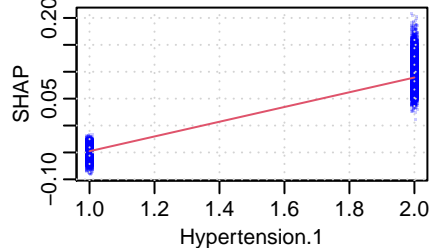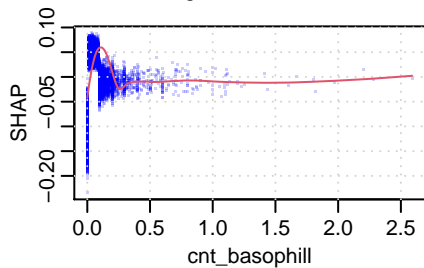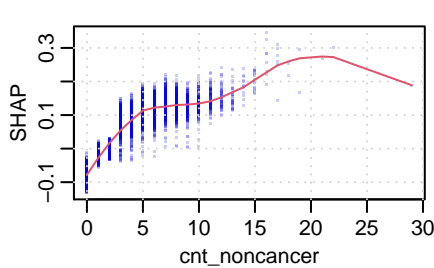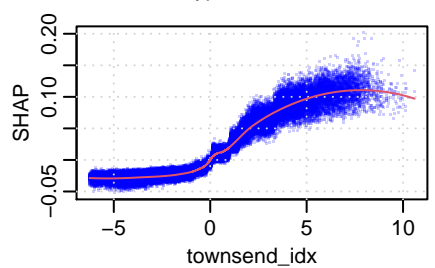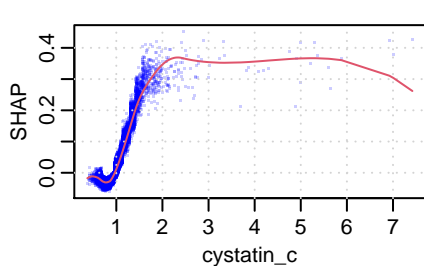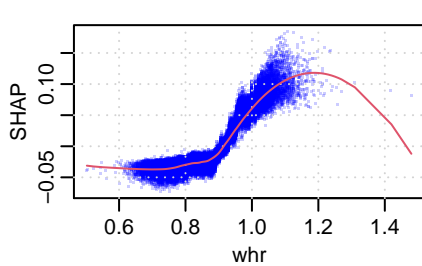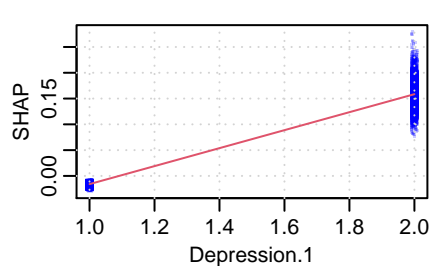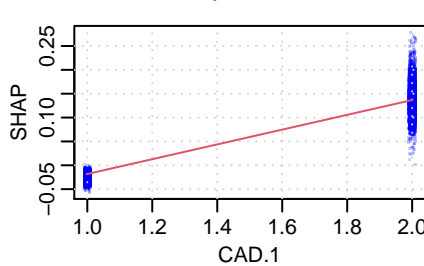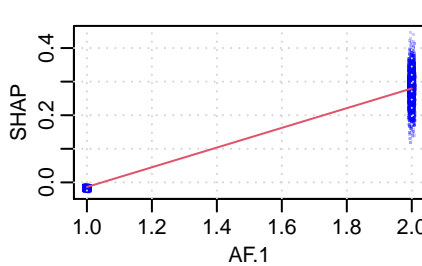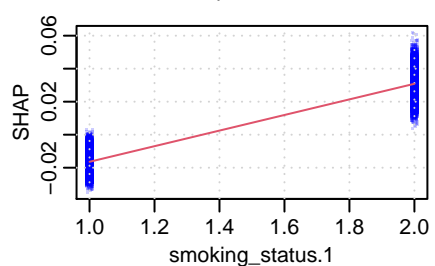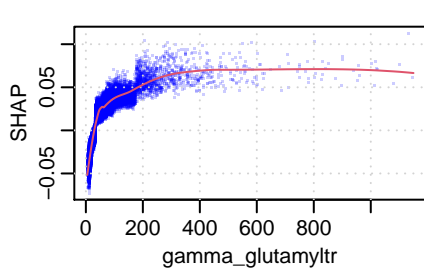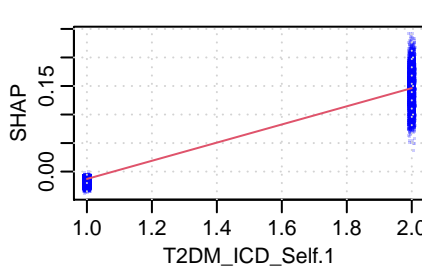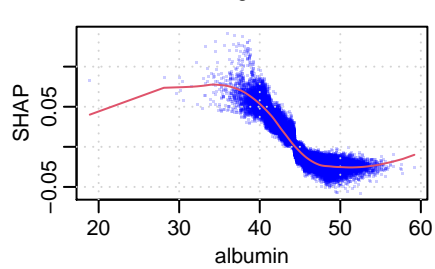

Supplement: Supplementary file 1 [file pharmaceutics-13-01514-s001.zip › Fig S1 xgboost_Pr(tested)_top15Variables_withLargest_abs_ShapleyValue.pdf]
